# Proportion of Idiopathic Pulmonary Fibrosis Risk Explained by Known Common Genetic Loci in European Populations

**DOI:** 10.1164/rccm.202008-3211LE

**Published:** 2021-03-15

**Authors:** Olivia C. Leavy, Shwu-Fan Ma, Philip L. Molyneaux, Toby M. Maher, Justin M. Oldham, Carlos Flores, Imre Noth, R. Gisli Jenkins, Frank Dudbridge, Louise V. Wain, Richard J. Allen

**Affiliations:** ^1^ University of Leicester Leicester, United Kingdom; ^2^ University of Virginia Charlottesville, Virginia; ^3^ Royal Brompton Hospital London, United Kingdom; ^4^ Imperial College London London, United Kingdom; ^5^ University of Southern California Los Angeles, California; ^6^ University of California Davis Davis, California; ^7^ Hospital Universitario Ntra. Sra. de Candelaria Santa Cruz de Tenerife, Spain; ^8^ Instituto de Salud Carlos III Madrid, Spain; ^9^ Instituto Tecnológico y de Energías Renovables Santa Cruz de Tenerife, Spain; ^10^ Instituto de Tecnologías Biomédicas (ITB), Universidad de La Laguna Santa Cruz de Tenerife, Spain; ^11^ University of Nottingham Nottingham, United Kingdom; ^12^ Nottingham University Hospitals Nottingham, United Kingdom and; ^13^ Glenfield Hospital Leicester, United Kingdom

*To the Editor*:

Understanding how genetic factors contribute to disease risk improves our understanding of pathogenesis, supports drug development, and aids risk prediction (1). Appropriate quantification and interpretation of this contribution is essential for measuring the impact of genetic variation and in motivating and informing future studies.

Idiopathic pulmonary fibrosis (IPF) is a chronic disease characterized by scarring of the lungs. Current therapies only slow disease progression and half of individuals die within 3–5 years of diagnosis. A genetic variant, rs35705950, in the *MUC5B* (mucin 5B) gene promoter region is strongly associated with IPF susceptibility with the risk allele (T) associated with a fivefold increase in disease risk (2). Genome-wide association studies (GWAS) have identified 13 additional independent IPF susceptibility variants (3).

The rs35705950_T allele frequency in IPF cases is 30–35% (4) (compared with 11% in controls), but risk allele frequency does not reflect the disease risk accounted for by this variant. Explained risk can be measured in different ways, such as the proportion of risk explained in the general population or, alternatively, the proportion of cases due to a specific variant.

Here we provide estimates of the proportion of IPF risk in the general population explained by known IPF susceptibility variants, and estimates of the proportion of cases attributable to each susceptibility variant. Our analyses focused on nonfamilial IPF; therefore, variants considered are just those evidenced by GWAS.

Some of the results of these studies have been previously reported in the form of a preprint (https://doi.org/10.1101/2020.08.14.20172528).

## Methods

We investigated the proportion of risk explained by the 14 IPF risk variants from a meta-analysis of previous IPF GWAS (3). To do this, we used unrelated European IPF cases (diagnosed according to international guidelines [5]) and controls, with appropriate ethics approval, that were used to replicate three signals in a previous study (namely, those near *DEPTOR*, *MAD1L1*, and *KIF15*, with the remaining 11 signals being replicated elsewhere [6–8]). These cases and controls were not used for the original discovery of any of the 14 variants as associated with IPF risk.

To estimate the proportion of disease risk explained by each variant in the general population, we performed regression analyses including the susceptibility variant as the only covariate. *R*^2^ is a measure of phenotypic variance explained by a model and, as our model only contains a single variant, the proportion of disease explained by that variant. *R*^2^ cannot be directly calculated as the IPF phenotype is binary and the proportion of cases in our analysis is higher than that observed in the general population. We therefore calculated a liability *R*^2^ accounting for enrichment of cases (9). The liability model assumes individuals have an unmeasured continuous trait, called the liability, and an individual develops IPF when the liability exceeds a critical value. We transformed the *R*^2^ to the liability scale and made an adjustment for ascertainment bias using the following equation:

(1) $$\text{Liability}\;R^{2}=\frac{R_{o}^{2}C}{1+R_{o}^{2}C\theta },$$

where $R_{o}^{2}$ is the coefficient of determination on the observed scale from a simple linear regression, and

(2) $$C=\frac{K\left(1-K\right)}{z^{2}}\frac{K\left(1-K\right)}{P\left(1-P\right)}$$

(3) $$\theta =m\frac{P-K}{1-K}\left(m\frac{P-K}{1-K}-t\right),$$

where *K* is the population prevalence, *P* is the proportion of cases in the study, *m* is the mean liability for cases, *t* is the liability threshold, and *z* is the normal density height at threshold *t*. We calculated the liability *R*^2^ for IPF prevalence estimates (i.e., *K* in the above equations) of 1.25 and 63 cases per 100,000 people (the lowest and highest reported estimates of disease prevalence in the general population [10]), and also using a disease prevalence of 495 cases per 100,000 people (the estimated disease prevalence in people >65 years of age [11]). To estimate the variance in the liability explained by all variants, we fitted the model with the most significant variants from all 14 known IPF susceptibility loci and calculated the liability *R*^2^. Finally, we fitted the model with all susceptibility variants, minus rs35705950. We investigated whether results were biased by population stratification by repeating analyses including 10 genetic principal components to adjust for ancestry.

To estimate the proportion of cases attributable to each variant, we calculated the population attributable risk fraction (12) (PARF). PARF is the proportion of cases that would be prevented if a risk factor were removed from the population. PARF can be calculated by

(4) $$\text{PARF}=1-\frac{1}{{\left(1-p\right)}^{2}+2p\left(1-p\right)e^{\beta }+p^{2}e^{2\beta }},$$

where *p* is the risk allele frequency in controls and β is the log(odds ratio) for the variant calculated using a simple logistic regression equation that includes the variant as the only covariate. We calculated 95% confidence intervals for PARF using parametric bootstrapping. If any risk factors were removed, the PARFs of other risk factors would change. Therefore, PARFs cannot be summed to calculate the proportion of cases prevented if multiple risk factors were removed.

## Results

**Figure 1.**
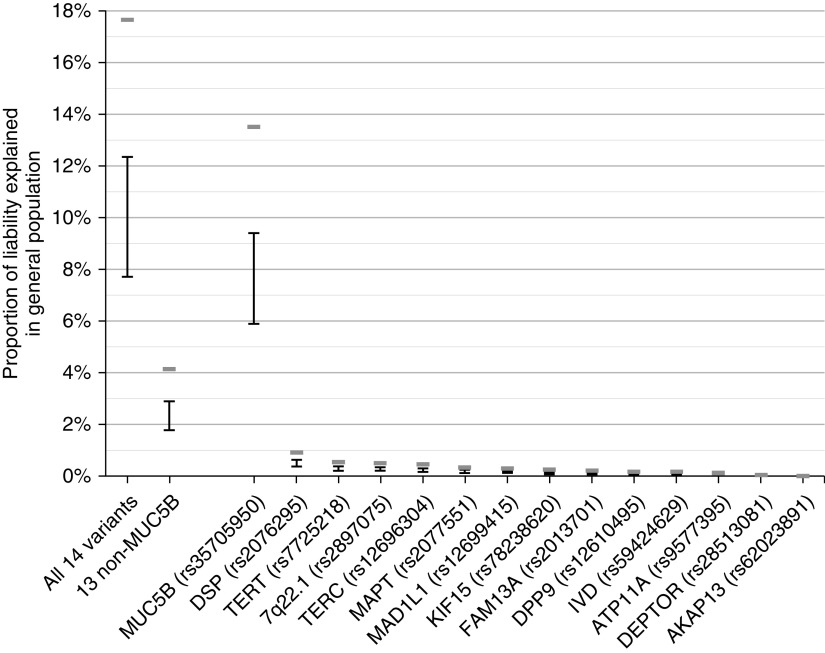
Proportion of liability explained in the general population. Estimated proportion of variation explained is taken from the liability *R*^2^ from the regression analyses with the lower bound given when assuming a disease prevalence of 1.25 cases per 100,000 and the upper bound given when assuming a disease prevalence of 63 cases per 100,000. The gray bars show the estimated proportion of liability explained when assuming the disease prevalence in people >65 years of age of 495 cases per 100,000. The *x*-axis label “All 14 variants” refers to the model including all 14 sentinel idiopathic pulmonary fibrosis (IPF) susceptibility variants; “13 non-MUC5B” refers to the model including all sentinel IPF susceptibility variants minus the *MUC5B* polymorphism rs35705950. Variants are ordered by the proportion of explained variation.

**Figure 2.**
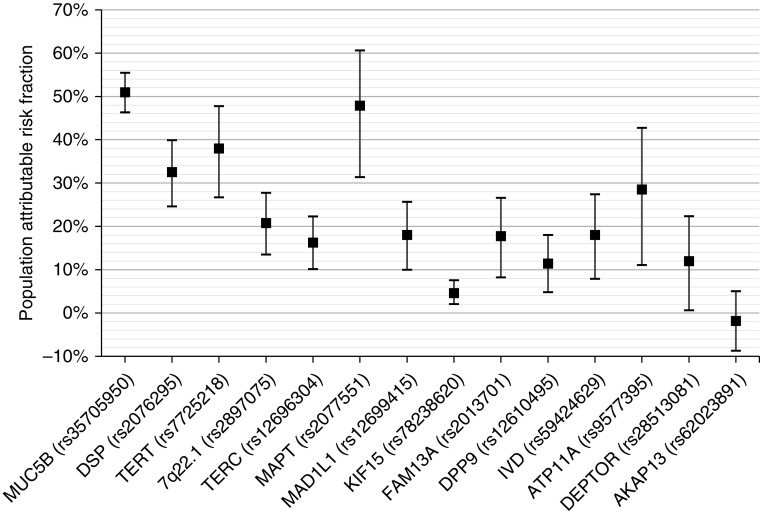
Population attributable risk fraction (PARF). Estimates of PARF are shown for each variant with 95% confidence intervals. Variants are ordered by the proportion of explained variation in the general population (Figure 1).

A total of 792 IPF cases and 10,000 controls were included in the analysis. Variant rs35705950 alone explains 5.9–9.4% of disease liability in the general population and 13.5% in people >65 years of age. No other IPF susceptibility variant explained more than 1% and collectively the 13 non-*MUC5B* susceptibility variants explained 1.8–2.9% of variation in disease liability in the general population and 4.2% in people >65 years of age (Figure 1). The highest PARF was observed for rs35705950 (51%); however, many of the susceptibility variants had PARF >10% (Figure 2). Effect sizes were similar after adjusting for principal components, suggesting that results are not biased by population stratification.

## Discussion

The *MUC5B* promoter polymorphism explains three times more disease liability (both in the general population and in people >65 yr of age) than the other 13 IPF susceptibility variants combined. In total, the 14 IPF susceptibility variants explain up to 12.4% of disease liability in the general population and 17.7% in people >65 years of age, which is smaller than previous reports that cited 30–35% of risk (4, 13). Importantly, however, therapies that target variant effects that explain a small proportion of disease risk can still have a large clinical impact (1).

Our results suggest IPF cases could be halved if the *MUC5B* risk allele was removed from the population. Although the clinical relevance of PARF estimates may be limited as removing risk alleles from the general population is almost impossible, they do indicate the impact preventive interventions could have on disease incidence.

Some IPF risk variants explain a small proportion of disease liability while having a high attributable risk. For example, the IPF risk allele rs2077551_T explains less than 0.4% variance in liability but has an attributable risk fraction of 47.9%. This is a consequence of the high frequency of the risk allele in the population (80.6%) with a relatively low odds ratio (OR) for disease (OR = 1.48). The variant rs62023891, near *AKAP13*, was not significantly associated with IPF in the particular data set used for these analyses and the effect estimate was close to zero and in the opposite direction (hence the point estimate of PARF <0% for this variant). This signal, which has been reported in independent studies (3, 8), is further supported by recent research demonstrating involvement of *AKAP13* in fibrogenesis and IPF risk (14).

Different populations experience diverse environmental exposures and have varying allele frequencies, affecting the proportion of risk explained by these variants and meaning these results may not be generalizable to non-European populations. This is especially true for the *MUC5B* variant, which shows large variation in allele frequencies across populations (minor allele frequency ≈ 1% in European populations compared with minor allele frequency <1% in populations with ancestries from East Asia or Africa [15]). We also only investigated known common IPF susceptibility variants, although previous studies suggest there could be many undiscovered genetic variants contributing to IPF risk (3), and we have not investigated epistasis or gene–environment interactions. This means overall IPF risk explained by genetics will likely be much higher than the 12.4–17.7% explained by the known variants.

This study used an ascertained case–control study design and made assumptions about disease prevalence. Ideally, a general population cohort, such as UK Biobank (16), would be used for these analyses. However, in UK Biobank there are few self-reported cases (*n* = 104) and cases defined using hospital episode statistics J84.1 codes do not genetically resemble clinically recruited cases (rs35705950_T allele frequency in these cases is 20%). Therefore, we restricted analyses to a study with clinically recruited cases. The study used was not used in the discovery of the IPF susceptibility variants (3), meaning the estimates of risk explained should not be subject to winner’s curse bias.

There are multiple ways of quantifying the risk explained by a genetic variant. For this study, we have focused on two measures: one to estimate the liability explained in the general population and another to estimate the proportion of cases attributable to each variant. A previous study that compared different methods to estimate the risk explained found these gave generally consistent results with differences due to different assumptions being made and by working on different scales (12). We could also consider absolute risk. Assuming disease prevalence is 63 cases per 100,000 and using the previously reported (2) effect size for the *MUC5B* risk allele (OR = 4.99), for every 100,000 individuals with the rs35705950_GG genotype, we would expect 30 to have IPF, whereas for every 100,000 individuals with the rs35705950_GT genotype, we would expect 152 to have IPF. Therefore, although rs35705950 is strongly associated with disease risk, most individuals carrying the risk allele will not develop IPF.

Although risk allele frequencies in cases can be of interest, they are not a measure of explained risk. Many of the known IPF susceptibility variants have a high PARF but individually explain a small overall proportion of the variation in risk. These results provide an important reference point to inform future genetic discoveries and for evaluation of the likely contribution of genetic factors in risk prediction models.

## References

[1] Zeggini E Gloyn AL Barton AC Wain LV Translational genomics and precision medicine: moving from the lab to the clinic *Science* 2019 365 1409 1413 31604268 10.1126/science.aax4588

[2] Zhu QQ Zhang XL Zhang SM Tang SW Min HY Yi L *et al* Association between the MUC5B promoter polymorphism rs35705950 and idiopathic pulmonary fibrosis: a meta-analysis and trial sequential analysis in Caucasian and Asian populations *Medicine (Baltimore)* 2015 94 e1901 26512610 10.1097/MD.0000000000001901 PMC4972586

[3] Allen RJ Guillen-Guio B Oldham JM Ma SF Dressen A Paynton ML *et al* Genome-wide association study of susceptibility to idiopathic pulmonary fibrosis *Am J Respir Crit Care Med* 2020 201 564 574 31710517 10.1164/rccm.201905-1017OC PMC7047454

[4] Seibold MA Wise AL Speer MC Steele MP Brown KK Loyd JE *et al* A common MUC5B promoter polymorphism and pulmonary fibrosis *N Engl J Med* 2011 364 1503 1512 21506741 10.1056/NEJMoa1013660 PMC3379886

[5] Raghu G Remy-Jardin M Myers JL Richeldi L Ryerson CJ Lederer DJ *et al* American Thoracic Society, European Respiratory Society, Japanese Respiratory Society, and Latin American Thoracic Society Diagnosis of idiopathic pulmonary fibrosis. an official ATS/ERS/JRS/ALAT clinical practice guideline *Am J Respir Crit Care Med* 2018 198 e44 e68 30168753 10.1164/rccm.201807-1255ST

[6] Noth I Zhang Y Ma SF Flores C Barber M Huang Y *et al* Genetic variants associated with idiopathic pulmonary fibrosis susceptibility and mortality: a genome-wide association study *Lancet Respir Med* 2013 1 309 317 24429156 10.1016/S2213-2600(13)70045-6 PMC3894577

[7] Fingerlin TE Murphy E Zhang W Peljto AL Brown KK Steele MP *et al* Genome-wide association study identifies multiple susceptibility loci for pulmonary fibrosis *Nat Genet* 2013 45 613 620 23583980 10.1038/ng.2609 PMC3677861

[8] Allen RJ Porte J Braybrooke R Flores C Fingerlin TE Oldham JM *et al* Genetic variants associated with susceptibility to idiopathic pulmonary fibrosis in people of European ancestry: a genome-wide association study *Lancet Respir Med* 2017 5 869 880 29066090 10.1016/S2213-2600(17)30387-9 PMC5666208

[9] Lee SH Goddard ME Wray NR Visscher PM A better coefficient of determination for genetic profile analysis *Genet Epidemiol* 2012 36 214 224 22714935 10.1002/gepi.21614

[10] Nalysnyk L Cid-Ruzafa J Rotella P Esser D Incidence and prevalence of idiopathic pulmonary fibrosis: review of the literature *Eur Respir Rev* 2012 21 355 361 23204124 10.1183/09059180.00002512 PMC9487229

[11] Raghu G Chen SY Yeh WS Maroni B Li Q Lee YC *et al* Idiopathic pulmonary fibrosis in US Medicare beneficiaries aged 65 years and older: incidence, prevalence, and survival, 2001-11 *Lancet Respir Med* 2014 2 566 572 24875841 10.1016/S2213-2600(14)70101-8

[12] Witte JS Visscher PM Wray NR The contribution of genetic variants to disease depends on the ruler *Nat Rev Genet* 2014 15 765 776 25223781 10.1038/nrg3786 PMC4412738

[13] Evans CM Fingerlin TE Schwarz MI Lynch D Kurche J Warg L *et al* Idiopathic pulmonary fibrosis: a genetic disease that involves mucociliary dysfunction of the peripheral airways *Physiol Rev* 2016 96 1567 1591 27630174 10.1152/physrev.00004.2016 PMC5243224

[14] Guillotin D Taylor AR Platé M Mercer PF Edwards LM Haggart R *et al* Transcriptome analysis of IPF fibroblastic foci identifies key pathways involved in fibrogenesis *Thorax*[online ahead of print] 19 Nov 2020; DOI: 10.1136/thoraxjnl-2020-214902 10.1136/thoraxjnl-2020-214902 33214245

[15] Auton A Brooks LD Durbin RM Garrison EP Kang HM Korbel JO *et al* 1000 Genomes Project Consortium A global reference for human genetic variation *Nature* 2015 526 68 74 26432245 10.1038/nature15393 PMC4750478

[16] Sudlow C Gallacher J Allen N Beral V Burton P Danesh J *et al* UK biobank: an open access resource for identifying the causes of a wide range of complex diseases of middle and old age *PLoS Med* 2015 12 e1001779 25826379 10.1371/journal.pmed.1001779 PMC4380465

